# Multimodal Therapy for the Treatment of Severe Ischemic Stroke Combining Endovascular Embolectomy and Stenting of Long Intracranial Artery Occlusion

**DOI:** 10.1155/2010/138023

**Published:** 2010-07-07

**Authors:** Matjaž Bunc, Igor J. Kocijančič, Rado Pregelj, Vinko V. Dolenc

**Affiliations:** ^1^Clinical Department for Cardiology, Zaloška cesta 2, University Clinical Centre Ljubljana, 1000 Ljubljana, Slovenia; ^2^International Institute for Neurosurgery and Neurosciences, Avcinova 12, 1000 Ljubljana, Slovenia

## Abstract

Embolic occlusion of cerebral arteries is a major cause for stroke. Intravenous thrombolysis showed positive results in this condition, however even when strict criteria are used, the risk of hemorrhagic transformation is possible. Microsurgical embolectomy has been described earlier. *Purpose*. We performed multimodal therapy of cerebral artery occlusion. *Case Report*. We present a case of a 49-year-old female patient who—according to the National Institute of Health Stroke Scale (NIHSS)—was rated as 19 due to acute occlusion of the horizontal segment of the left middle cerebral artery (MCA). After failed i.v. thrombolysis, only a part of the clot could be evacuated by the endovascular approach—without restoration of blood flow. Normal patency of the left MCA was re-established after stenting. Within 72 hours, the patient had an NIHSS score of 14, with a small haematoma in the left hemisphere. *Conclusion*. In our case multimodal therapy combining i.v. thrombolysis, mechanical disruption of thrombus, MCA stenting and platelet function antagonists, resulted in successful recanalization of the acutely occluded left MCA.

## 1. Introduction

A large majority of cardioembolic and atherosclerotic artery-to-artery thromb-emboli share architectural features of random fibrin: platelet deposits interspersed with linear collections of nucleated cells (monocytes and neutrophils) and confined erythrocyte-rich regions. The thromboembolic structure provides a foundation for both antiplatelet and anticoagulant treatment strategies in stroke prevention and treatment [1]. 

Intraarterial and intravenous thrombolysis are however often ineffective for the treatment of acute ischemic stroke. Even when strict criteria are applied, the risk of hemorrhagic transformation is present. The possibility of microsurgical thromb-embolectomy has been described already three decades ago [2–10], and recently endovascular mechanical thromb-embolectomies were added in addition to microsurgical thromb-embolectomies practised prior to endovascular modality [11]. In the endovascular mechanical Embolus Removal in Cerebral Ischemia (MERCI) study [12], a phase 1 trial shows that MCA endovascular embolectomy with the MERCI Retriever [12] was safe and that successful recanalization could benefit a significant number of patients, even when performed in on extended 8-hour time window [13]. However, a model of the internal carotid artery (ICA) balloon embolectomy for ICA thrombosis demonstrated risks of further embolic cerebral damage [14]. Therefore a multimodal rescue therapy combining thrombolysis, mechanical disruption of the clot, stenting and platelet function antagonists may improve re-canalization of on acutely occluded intracranial artery.

## 2. Patient and Methods

**Figure 1:**
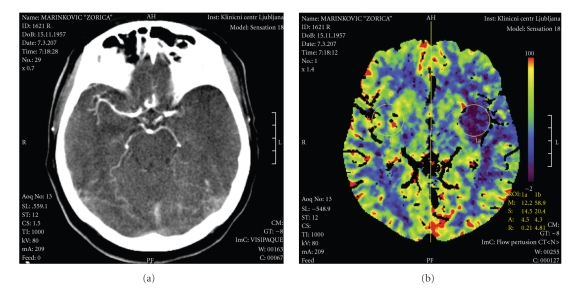
(a) Contrast-enhanced cranial CT with discontinuation of left MCA. (b) CT perfusion test revealed increased time to peak in the velocity.

**Figure 2:**
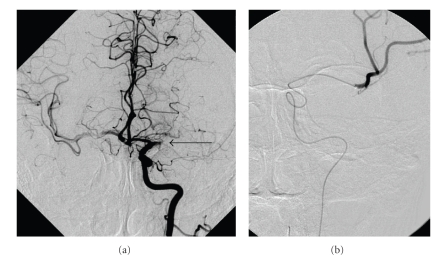
(a) Diagnostic angiogram (left internal carotid artery injection, anteroposterior projection) shows (arrow) the occlusion of the horizontal segment of the left middle cerebral artery (MCA). (b) Coaxial catheterization with a microcatheter and stirable microguidewire entering the occluded vessel, traversed the occluded segment of the left MCA. With the contrast, the peripheral MCA is visualized.

A 49-year-old female patient presented with the NIHSS score of 19 (right-sided complete hemiplegia and aphasia) one hour after the onset of symptoms and signs. Urgent CT scan (Figure 1(a)) demonstrated a hyperdense signal in the proximal segment of the left MCA (Hyperdense Middle Cerebral Artery Sign—HMCAS) and CT perfusion test revealed increased time to peak in the velocity (Figure 1(b)). Since the i.v. thrombolysis failed, urgent digital subtraction angiography (DSA) was performed (Figure 2(a)). 

Occlusion of the horizontal segment of the left MCA was demonstrated. (Figure 2(a)) Despite the early signs of ischemia it was decided to try endovascular thromb-embolectomy.

A regular angiographic technique was used for the intervention. In our setting, this technique includes the use of a 6F introducer in the femoral artery through which a 6F guiding catheter was inserted into the extracranial segment of the left ICA into the occluded left MCA. Then followed coaxial catheterization with a microcatheter and stirable microguidewire entering the occluded left MCA and then the microcatheter was positioned with its tip just distal to the occlusion, which was proven with the injection of the contrast media, and the “normal” arterial outline was visualized peripheral to the embolus. (Figure 2(b)).

**Figure 3:**
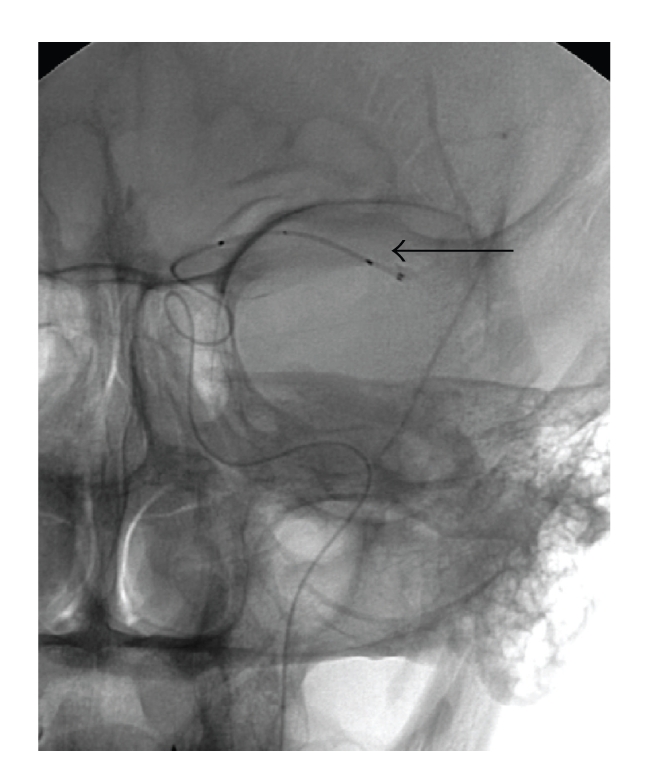
Vascular retrieval snare in the distal part of microcatheter. The snare (arrow) has been deployed out of the microcatheter just enough to open fully, and retrieved back in the microcatheter.

A vascular retrieval snare (Amplatz Goose Neck; Microvena Corporation, White Bear Lake, MN) is then introduced (Figure 3). 

**Figure 4:**
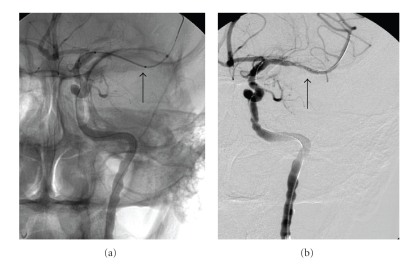
Angiogram before and after deployment of the intraarterial Neuroform Stent 3 × 20 mm (arrows).

Endovascular mechanical thrombolectomy with retriever was attempted several times and only a part of the clot was retrieved, without restoration of blood flow. It was then decided to place a stent. An intracranial stent (Boston Scientific, Neuroform 3) 3 × 20 mm (Figure 4(a)) was placed in the horizontal MCA with sufficient restoration of flow (Figure 4(b)). 

**Figure 5:**
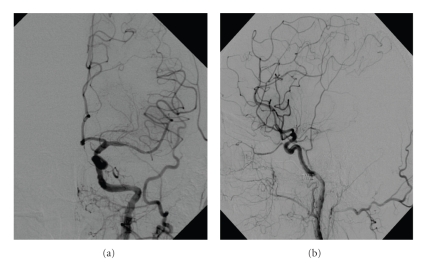
Follow-up angiogram immediately after re-opening of the left MCA (left ICA injection), anteroposterior (a) and lateral (b) views show a patent, though slightly narrower left MCA (arrows).

Control angiography immediately after stenting showed patent the left MCA in A-P projection (Figure 5).

**Figure 6:**
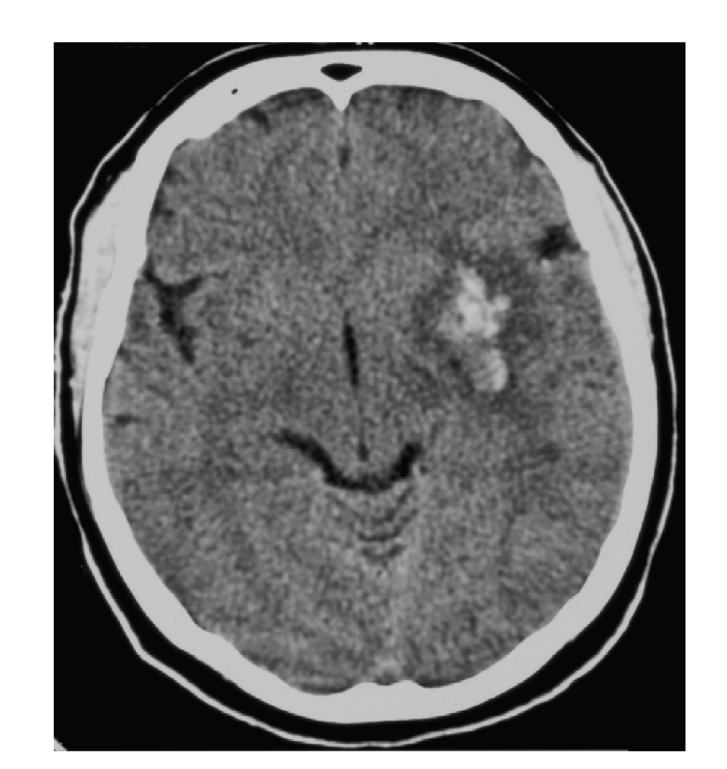
Control CT scan shows a small haematoma in the left hemisphere.

Within 72 hours, the patient had an NIHSS score improved to 14, with a small haematoma in the left hemisphere (Figure 6); TCD demonstrated normal flow in the left MCA artery.

**Figure 7:**
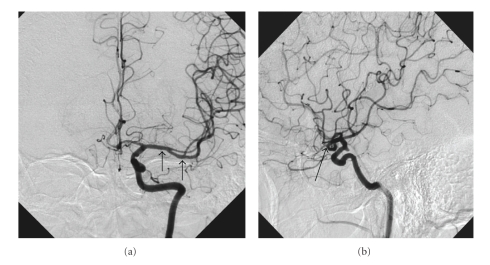
Follow-up angiograms—antero-posterior (a) and lateral views (b) 6 months later. The stented segment of MCA is indicated with arrows.

Six months after treatment: thrombolytic therapy and stenting of the left MCA angiogram shows good patency of the stented artery (Figures 7(a) and 7(b)). Restoration of blood flow in the other M2 braches of MCA is visualized. There is also improvement in the clinical neurological status with only slight residual right hand paresis.

## 3. Discussion

Stroke usually causes neurological disability to the patient. An early recanalization of the acutely occluded artery is very important. Direct surgical arteriotomy with thromb-embolectomy of the MCA was performed already in 1955 [9]. Several years later (1961), when microsurgery was introduced, Jacobson et al., used magnification at such operations [6, 8]. In 1962, Yasargil reported already 11 cases of direct arteriotomy of the MCA, in which thromb-embolectomies were performed, and the patency of the arteries was re-established [10]. Toward the end of the 80s, 40 cases of surgical recanalizations of cerebral arteries were reported and in some cases with excellent results [3]. At that time already, Donaghy was of the opinion that incomplete occlusion of a cerebral artery does not require surgical intervention [4]. On the other hand, acute complete occlusion of the major cerebral artery which brings about a complete ischemia is most probably a definite situation. Several authors were of the opinion that even the fastest surgical intervention fails to be successful. Very interesting is Yasargil's observation, in which he had moderate improvement in a case operated 40 minutes after stroke and surprisingly good improvement in a patient subjected to surgery 5 months after stroke [10]. These two extremes do demonstrate big differences between individual cases. Also the animal studies of thromb-embolectomies of the MCA were not consistent regarding the time interval after the stroke: between occurrence of stroke and thromb-embolectomy [5]. Galibert, according to his own experience in treatment of MCA occlusion, was of the opinion that surgical treatment of MCA occlusion was far from being a systematic surgical indication [7]. On the basis of previous reports, Dechaume et al. were oriented pro- surgically in management of MCA occlusion [2]. They were of the opinion that good results of surgical trombectomies depend on: the side and extent of the arterial occlusion; cross-circulation with or without other anasthomosis; time elapsed from stroke to operation; age of patient; primary cause of embolism or thrombosis; surgical technique and performance of the operation.

With great advancement of endovascular interventions for numerous pathological conditions in the cerebral arteries, beside the intravenous thrombolysis, also endovascular thromb-embolectomy and finally stenting of the occluded major cerebral artery have become possible.

Studies have been performed to evaluate the effectiveness of i.v. thrombolysis in these condition [15–17]. Although these studies show positive results when strict criteria are applied, the risk of hemorrhagic transformation is possible, possibly because of the fibrinolytic agent. The use of a vascular retrieval snare intended for extracting foreign bodies from the vascular tree has already been described [15–17].

The result of the MERCI 1 trial (Mechanical Embolus Removal in Cerebral Ischemia) shows that endovascular embolectomy of the occluded cerebral artery with the MERCI Retriever was safe and successful. In our case, endovascular mechanical thromb-embolectomy with retriever was attempted several times without restoration of blood flow.

The MERCI Retriever evaluated by using experimental models does not always retain the embolism, and the lumen is rather rough [11]. The manipulation of a clot represents a high risk of its dislodgment or rupture and risks for peripheral embolisms. The prevalent histological structure, with a fibrin/platelet pattern of both cardio embolic and artery-to artery atherosclerotic thromboemboli that cause acute stroke, provides a foundation for both antiplatelet and anticoagulant treatment strategies in stroke prevention [1]. Therefore stenting of an occluded artery with additional antiplatelet therapy may be an appropriate option for reestablishment of arterial patency and sufficient cerebral blood flow.

Local intra-arterial thrombolysis (LIAT) was risky in our case since systemic i.v. thrombolysis was performed before intervention. It would have been optimal and safer if, after HMCAS was detected, LIAT were tried as the first method of choice. In this case LIAT might be an option of procedure optimalisation in case of distal embolization. 

In our case early restoration of the MCA flow was proven to be successful both in angiographic and in clinical status.

## 4. Conclusions

Early restoration of blood flow without exposing the patient to large doses of fibrinolytic drugs is an appealing prospect. In our case multimodal therapy combining i.v. thrombolysis, mechanical disruption of thrombus, MCA stenting and platelet function antagonists, resulted in successful recanalization of the acutely occluded left MCA.

Surgical treatment, which does offer an additional option for recanalization of the occluded artery should still be kept in mind, at least in centers where good coordination between the departments exists, and if sufficient surgical skill(s) are available.
